# A new 3D heterometallic coordination polymer: luminescent property and nursing application value on acute cerebral infarction

**DOI:** 10.1080/15685551.2022.2033431

**Published:** 2022-02-02

**Authors:** Li Cong, Yun-Zhi Pan, Ying-Hui Qin, Ji-Na Jiang

**Affiliations:** Department of Neurology Ward 1, The Third Affiliated Hospital of Qiqihar Medical University, Qiqihar, China

**Keywords:** Heterometallic compound, luminescence, acute cerebral infarction

## Abstract

With the solvothermal reactions of flexible tetracarboxylic acid ligand with the Cd(II) and Ca(II) ions, we acquired a new heterometallic coordination polymer formulated as {[Cd_2_Ca_2_(L)_2_(DMF)_2_(H_2_O)_7_]·(DMF)·2(H_2_O)}_n_ (**1**, H_4_L is 5-(bis(4-carboxybenzyl)amino)isophthalic acid, DMF is N,N’-Dimethylformamide). Furthermore, the solids of **1** shows ligand-centered luminescence at room temperature. It not only evaluated the treatment and nursing application value on acute cerebral infarction, but also explored the related mechanism. Above of all, ELISA assay measured the content of the MMP-9 released into the cerebrospinal fluid, and the real time RT-PCR was implemented and the NF-κB activation in the brain tissue was measured.

## Introduction

Acute cerebral infarction is an acute disease threatening the life safety of patients. Thrombolytic therapy is mainly used in the rescue process [1]. It ensures the normal blood supply by effective thrombolytic therapy, thereby improving ischemia and hypoxia, and reducing the acute brain as much as possible [2]. Thrombolytic therapy has a certain effect, however, some patients will experience cerebral hemorrhage during the period of treatment, and cerebral hemorrhage will make a further effect on the patient’s life safety, combined with an increased level of inflammatory response [3].

Metal-organic polymeric materials, namely coordination polymer (CPs), have experienced an explosive development owing to their fascinating structures, as well as on account of their potential application properties of magnetism, luminescence, adsorption/separation, photocatalysis, biomedicine, and so forth [4–7]. The construction of polymeric materials is through the coordination bond-driven self-assembly of metal ions and organic ligands under the conditions of hydro(solvo)thermal [8–11]. The architectures and performances of CPs have intimate correlation with organic building blocks. Therefore, selecting a suitable organic ligand for the construction of CPs with desired structures and properties is crucial. On this matter, multidentate organic polycarboxylic acids with π-electron conjugated aromatic benzene rings have been demonstrated to be appropriated organic building blocks for preparing the functional CPs not only owing to their architectural stability but also because of their abundant coordination modes to the metal centers [12–15]. H_4_L, as a flexible polycarboxylate ligand, has three π-electron conjugated aromatic benzene rings and four carboxylate groups that may enable it to bridge metal centers into topologically unique architectures with interesting luminescent properties [16]. In the current paper, it was chosen as organic ligand for the assembly of the dual metal ions of Cd(II) and Ca(II) under the conditions of solvothermal. Successfully, we acquired a new heterometallic compound of {[Cd_2_Ca_2_(L)_2_(DMF)_2_(H_2_O)_7_]·(DMF)·2 (H_2_O)}_n_ (**1**, H_4_L is 5-(bis(4-carboxybenzyl)amino)isophthalic acid, DMF is N, N’-dimethylformamine). The X-ray diffraction investigation indicated that the **1ʹ**s 3-dimensional structure features two different cluster-based building subunits: trinuclear [CdCa_2_(COO)] clusters and pentagonal [Cd_3_Ca_2_(COO)_4_] clusters, and represents a new unprecedented (4,12)-linked topological net containing {4^24^.6^34^.8^8^}{4^4^.6^2^}{4^5^.6}_4_ point symbol. It also investigated thermostability and luminescent property. The ELISA assay and real time RT-PCR were used to measure the treatment and nursing application value of the new compound on acute cerebral infarction in the biological section.

## Experimental

### Materials and instrumentation

The employed chemical reagents in the current experiment are acquired from market source and utilized directly. An Vario EL III elemental analyzer was employed for conducting the N, H together with C elemental analysis. A PANalytical X’Pert Pro with 1.54056 Å Cu/Kα radiation with 0.05° step size was applied for PXRD. TGA was implemented on a NETSCHZ STA-449C with 10 °C/min heating rate under the nitrogen flow between 30 and 800°C. A FLS920 luminescence spectrophotometer was utilized to explore the luminescent properties.

### Synthesis of {[Cd_2_Ca_2_(L)_2_(DMF)_2_(H_2_O)_7_]·(DMF)·2 (H_2_O)}_n_ (1)

The mixture of 0.100 mmol Cd(NO_3_)_2_ **· **4H_2_O, 0.100 mmol Ca(NO_3_)_2_ **· **4H_2_O, 0.1 mmol H_4_L, 3.0 mL DMF and 1.0 mL H_2_O was stored into a small glass vial (20 mL), and after that, at 110 °C in an oven the glass vial was sealed and heated for 2 days. The compound’s colorless massive crystals were gathered with 38% yield in the light of Cd(NO_3_)_2_ · 4H_2_O after the temperature of oven was cooled to RT. Elemental analysis calcd. for the C_57_H_67_Ca_2_Cd_2_N_5_O_28_ (1575.08): C, 43.46; H, 4.29; N, 4.45%. Found: C, 43.42, N, 4.43, and H, 4.27%.

### X-ray structural determination

On a computer-controlled Rigaku Mercury CCD diffractometer that equipped with graphite-monochromated Mo-Kα radiation, the crystal structural data of **1** was collected at RT. The direct mean together with the full-matrix least squares were respectively applied for the synthesis and modification of **1ʹ**s architecture with SHELXTL [17]. All hydrogen atoms were generated in their ideal positions, as well all non-hydrogen atoms were refined anisotropically. The PLATON program was used to squeeze out the lattice water molecules of **1**, and their detailed information has been determined via a combination of elemental analysis and TGA measurements. In Table 1, the crystallographic data and structural refinements of **1** were summarized, and the related bond parameters around Cd(II) and Ca(II) ions are listed.

**Table 1.** The **1ʹ**s crystal data

Formula	C_57_H_67_Ca_2_Cd_2_N_5_O_28_
Fw	1575.08
Crystal system	Monoclinic
Space group	*C*2/c
*a* (Å)	19.9688(11)
*b* (Å)	20.6166(8)
*c* (Å)	35.803(2)
*α*(°)	90
*β*(°)	105.457(3)
*γ*(°)	90
Volume (Å^3^)	14,206.4(13)
*Z*	8
Density (calculated)	1.439
Abs. coeff. (mm^−1^)	0.821
Total reflections	38,457
Unique reflections	14,817
Goodness of fit on *F^2^*	1.084
Final *R* indices [*I* > 2sigma(*I*^2^)]	*R* = 0.0512, *wR*_2_ = 0.1425
*R* (all data)	*R* = 0.0633, *wR*_2_ = 0.1497
CCDC	2142414

### ELISA assay

After the treatment of compound, the ELISA assay was conducted in this research to measure the content of the MMP-9 released into the cerebrospinal fluid. Under the guidance of the instructions with some modifications, this preformation was conducted totally. 50 C57 mice (4–5 weeks, 20–22 g) were used in this research. After utilizing the electrocoagulation to block the bilateral vertebral arteries in mice for 24 hours, the bilateral common carotid arteries were employed to induce acute cerebral infarction animal model. Afterwards, the animal was injected with compound at 1, 2 and 5 mg/kg concentration. The Animal Ethics Committee of China approved all the preformation in this experiment. After of all, the cerebrospinal fluid was collected as well as the content of inflammatory cytokines was determined with ELISA detection kit.

### Real time RT-PCR

To detect the NF-κB activation in brain tissue, and the novel compound’s influence on the NF-κB activation in brain tissue, the real time RT-PCR was performed in this paper for assessment. In consistence with the protocols with only a little change, this experiment was carried out strictly. Briefly, the electrocoagulation was used to block the bilateral vertebral arteries in mice for 24 hours, and then to induce acute cerebral infarction animal model, the bilateral common carotid arteries was utilized. After that, the compound was injected into the animal at 1, 2 and 5 mg/kg concentration. The total RNA in the cells was extracted with TRIZOL reagent and the astrocytes were collected. Then it was reverse transcript into cDNA after measuring the overall RNA concentration. Eventually, the real time RT-PCR was carried out, and the NF-κB activation in the brain tissue was determined.

## Results and discussion

### Crystal structure of compound 1

**Figure 1.**
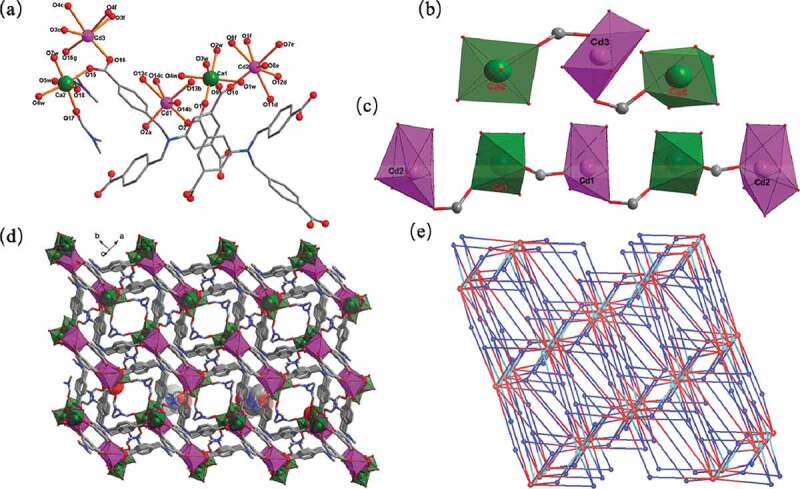
(a) The coordination surroundings of Ca(II) ion and Cd(II) ion in compound **1**. (b) The trinuclear [CdCa_2_(COO)_2_] cluster. (c) The pentagonal [Cd_3_Ca_2_(COO)_4_] cluster. (d) The compound’s 3-dimensional framework. (e) Schematic representation (4,12)-connected topological network for **1.**

The compound’s asymmetric unit is constituted by two Ca(II) ions, two Cd(II) ions, two coordinated molecules of DMF, two L^4-^, seven coordinated water molecules, one as well as one free DMF molecules and two disordered lattice water molecules as revealed via the elemental analysis and TGA measurements, and the structure of **1** indicates a 3-dimensional skeleton with (4,12)-bridged topology which is confirmed through the analysis of X-ray diffraction. Cd1 ion and Cd3 ion with 0.5 occupancy are 6-coordinated and exhibit twisted octahedral coordination structures which are reflected in Figure 1a. They are defined through six carboxylic acid oxygen atoms belonging to four diverse L4-, while Cd2 are seven-coordinated with a mono-capped triangular prism geometry defined through seven carboxylic acid oxygen atoms provided by four separated L^4-^. The Ca1 center and Ca2 center are in the center of distorted octahedrons. For Ca1, four terminal water ligands together with two carboxylate oxygen atoms come from two distinct L^4-^ surrounded the octahedrally coordinated Ca1 centers. For Ca2, two terminal DMF ligands, three terminal water ligands, as well as a carboxylic acid oxygen atom derived from a L^4-^ offered its octahedral coordination environments. All the separations of Ca-O and Cd-O bond listing in Table S1 are comparable with those of the past reports [18]. The flexible tetracarboxylic acid ligand of H_4_L is completely deprotonated, and shows two different coordination modes in the construction of polymer **1**, which can be described as (*κ*^1^-*κ*^1^)-(*κ*^2^)-(*κ*^2^)-(*κ*^1^-*κ*^1^)-*μ*_6_ and (*κ*^1^-*κ*^1^)-(*κ*^2^)-(*κ*^2^)-(*κ*^2^)-*μ*_5_ (Fig. S1). It is fascinating that two diverse heterometallic clusters are generated through the linkage of carboxylic acid groups with heterometal centers. As exhibited in Figure 1b and c, two bis-monodentate carboxylic acid groups link two Ca2 ions and one Cd3 ion to form the heterometallic trinuclear cluster, and four bis-monodentate carboxylic acid groups connect a Cd1 ion, two Cd2 ions and two Ca1 ions into a heterometallic pentanuclear cluster. The L^4-^ ligands, extending into a 3-dimensional framework (Figure 1d) connected these trinuclear and pentanuclear heterometallic clusters. Topological analysis was applied to simplify the complicated frameworks of polymeric MOFs, which was implemented in order to get a better insight of this complicated 3-dimensional skeleton, and the analysis outcome reflects that the L^4-^ ligands, trinuclear and pentanuclear heterometallic clusters can be simplified topologically into 4, 4, 12-linked nodes, respectively. Hence, such complex framework can be considered as a (4,12)-linked trinodal topological net with {4^24^.6^34^.8^8^}{4^4^.6^2^}{4^5^.6}_4_ point symbol (Figure 1e).According to our understanding, such topological network reported here in the area of MOFs which has not been reported before.

### Powder X-ray diffraction pattern (PXRD) and thermogravimetric analysis (TGA)

**Figure 2.**
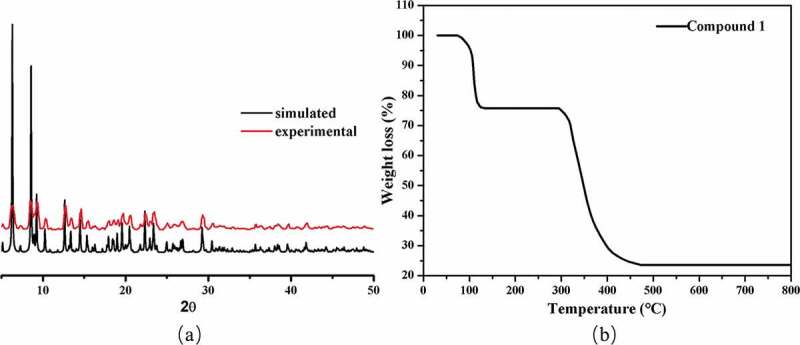
(a) The compound’s PXRD modes. (b) and its curve of TGA.

As exhibited in Figure 2a, the experimental pattern according to the massive samples in accordance well with pattern simulated from the data of single crystal architecture, confirming its high phase purity.

The TGA experiment investigated the heat resistance of **1** under a nitrogen atmosphere. The TGA result is plotted in Figure 2b. Because of the release of coordinated and free solvent molecules (Calcd: 24.20%), in the TGA curve and from 74 to 135 °C, a weight loss of 24.26% can be observed. The skeleton of **1** after loss the solvent molecules shows no weight loss before 295 °C. After that, the framework started to quickly collapse on account of the organic ligand decomposition.

### Luminescent property of compound 1

**Figure 3.**
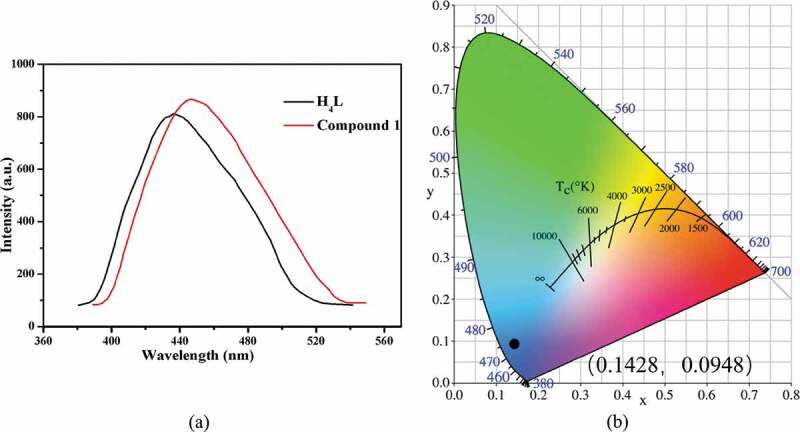
(a) The solid-state emission spectra of **1** and free H_4_L ligand at room temperature. (b) The CIE chromaticity diagram for **1.**

Coordination polymers have drawn tremendous attention owing to their excellent luminescent properties that can be used in sensing, light-emitting diodes, and so on [19,20], which assembled from d^10^ transition metal ions and organic ligands with large conjugated groups. The luminescent properties of **1** and free H_4_L ligand were investigated at room temperature after considering that. When excited by 350 nm, compound **1** exhibits a broad emission band with the maxima peak at 446 nm (Figure 3a). Upon excitation of 360 nm, the free H_4_L ligand has an emission peak at 436 nm (Figure 3a), which can be assigned to π*→π transitions of π-electron conjugated benzene rings [21]. The emission peak of **1** has a red-shift of 10 nm relative to that of H_4_L ligand. Owing to the inoxidability or irreducibility of Ca(II) and Cd(II) ions, the luminescent property of **1** is neither ligand-to-metal charge transfer (LMCT) nor metal-to-ligand charger transfer (MLCT) [22–24]. The luminescence of **1** is mostly derived from intraligand charge transfer of H_4_L according to the reports. The CIE chromaticity coordinate of **1** is calculated at (0.1428, 0.0948) (Figure 3b), which indicating that compound **1** may be acted as a good blue luminescent material.

### Compound significantly reduced the content of the MMP-9 released into the cerebrospinal fluid

**Figure 4.**
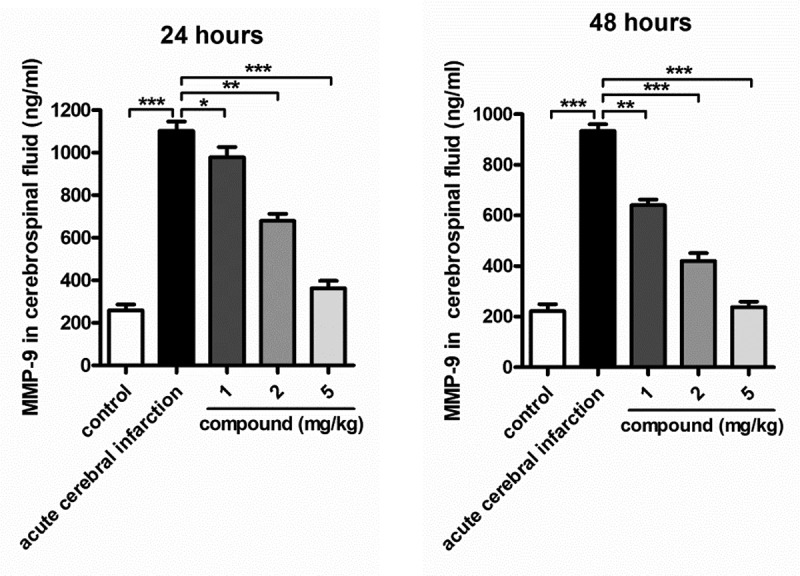
Significantly reduced content of the MMP-9 released into the cerebrospinal fluid after compound treatment. The acute cerebral infarction model was induced, then the new compound was given for treatment at the concentration of 1, 2 and 5 mg/ml. The ELISA assay measured the content of the MMP-9 released into the cerebrospinal fluid.

In this research, after the acute cerebral infarction model construction and the treatment of compound, the content of the MMP-9 released into the cerebrospinal fluid was evaluated firstly. So, in this experiment, the ELISA assay was conducted. In Figure 4, it shows that there was a higher level of the content of the MMP-9 released into the cerebrospinal fluid in the model group, which is not apparently same as the control group, with P < 0.005. After the new compound treatment, the content of the MMP-9 released into the cerebrospinal fluid was decreased. The inhibition of the new compound exhibited in a dose-dependent manner.

### Compound obviously inhibited the activation of the NF-κB in the brain tissue dose dependently

**Figure 5.**
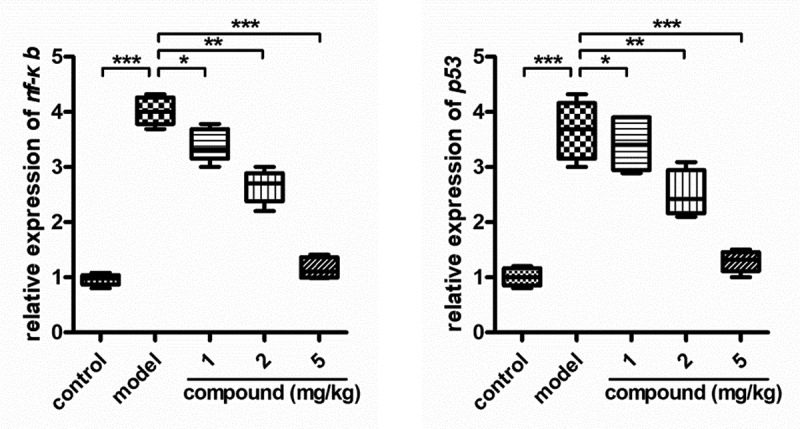
Obviously inhibited activation of the NF-κB in the brain tissue dose dependently after compound treatment. The acute cerebral infarction model was induced, then the new compound was given for treatment at the concentration of 1, 2 and 5 mg/ml. The activation of the NF-κB in the brain tissue was further determined with real time RT-PCR assay.

In the research of above, the compound could obviously reduce the content of the MMP-9 released into the cerebrospinal fluid in a dose dependent manner was proved. Furthermore, the NF-κB activation in the brain tissue could also regulate the content of the MMP-9 released into the cerebrospinal fluid. So, the real time RT-PCR assay determined the activation of the NF-κB in the brain tissue furtherly. In Figure 5, we can see that the activation of the NF-κB in the brain tissue of the model group was higher than that of the control group. There was a significant difference between these two groups. Under the new compound treatment, the activation of the NF-κB in the brain tissue was obviously reduced. The inhibition of the new compound showed a dose-dependent manner.

## Conclusion

In summary, under solvothermal conditions, a new 3D heterometallic coordination polymer of [Cd_2_Ca_2_(L)_2_(DMF)_2_(H_2_O)_7_]_n_·n(DMF)·2 n(H_2_O) (**1**) was successfully synthesized. The 3D framework of **1** contains two different heterometallic cluster-based subunits: trinuclear [CdCa_2_(COO)] clusters and pentagonal [Cd_3_Ca_2_(COO)_4_] clusters, and can be simplified into an unprecedented (4,12)-connected topological network. Compound **1** can be served as a potential photoactive material which is indicated by the intense luminescence. The results of the ELISA assay indicated that the compound could obviously reduce the content of the MMP-9 released into the cerebrospinal fluid. With the exception of this, the new compound dose dependently inhibited the activation of the NF-κB in the brain tissue also.

## Data Availability

Selected bond lengths (Å) and angles (^°^) for **1** (Table S1); The coordination modes of L^4-^ ligand in **1** (Fig. S1), the information could be found in the supporting information file.
